# PI3K pan-inhibition impairs more efficiently proliferation and survival of T-cell acute lymphoblastic leukemia cell lines when compared to isoform-selective PI3K inhibitors

**DOI:** 10.18632/oncotarget.3295

**Published:** 2015-03-16

**Authors:** Annalisa Lonetti, Alessandra Cappellini, Antonino Maria Spartà, Francesca Chiarini, Francesca Buontempo, Camilla Evangelisti, Cecilia Evangelisti, Ester Orsini, James A. McCubrey, Alberto Maria Martelli

**Affiliations:** ^1^ Department of Biomedical and Neuromotor Sciences, University of Bologna, Bologna, Italy; ^2^ Department of Human, Social and Health Sciences, University of Cassino, Cassino, Italy; ^3^ Muscoloskeletal Cell Biology Laboratory, IOR, Bologna, Italy; ^4^ Institute of Molecular Genetics, National Research Council-Rizzoli Orthopedic Institute, Bologna, Italy; ^5^ Department of Microbiology and Immunology, Brody School of Medicine, East Carolina University, Greenville, NC, USA

**Keywords:** PI3K isoforms, PTEN, T-ALL, targeted therapy, autophagy

## Abstract

Class I phosphatidylinositol 3-kinases (PI3Ks) are frequently activated in T-cell acute lymphoblastic leukemia (T-ALL), mainly due to the loss of PTEN function. Therefore, targeting PI3Ks is a promising innovative approach for T-ALL treatment, however at present no definitive evidence indicated which is the better therapeutic strategy between pan or selective isoform inhibition, as all the four catalytic subunits might participate in leukemogenesis. Here, we demonstrated that in both *PTEN* deleted and *PTEN* non deleted T-ALL cell lines, PI3K pan-inhibition exerted the highest cytotoxic effects when compared to both selective isoform inhibition or dual p110γ/δ inhibition. Intriguingly, the dual p110γ/δ inhibitor IPI-145 was effective in Loucy cells, which are representative of early T-precursor (ETP)-ALL, a T-ALL subtype associated with a poor outcome. *PTEN* gene deletion did not confer a peculiar reliance of T-ALL cells on PI3K activity for their proliferation/survival, as PTEN was inactivated in *PTEN* non deleted cells, due to posttranslational mechanisms. PI3K pan-inhibition suppressed Akt activation and induced caspase-independent apoptosis. We further demonstrated that in some T-ALL cell lines, autophagy could exert a protective role against PI3K inhibition. Our findings strongly support clinical application of class I PI3K pan-inhibitors in T-ALL treatment, with the possible exception of ETP-ALL cases.

## INTRODUCTION

Class I phosphatidylinositol 3-kinases (PI3Ks) comprise members of a conserved family of heterodimeric intracellular lipid kinases involved in crucial aspects of cell growth and survival through phosphorylation of phosphatidylinositol-4, 5-bisphosphate (PIP_2_) to phosphatidylinositol-3, 4, 5-trisphosphate (PIP_3_) which acts as an intracellular second messenger by binding with high affinity to pleckstrin homology (PH) domains in target molecules [1]. Among the binding partners, there are the serine-threonine protein kinases Akt and phosphoinositide-dependent kinase 1 (PDK1). Recruitment to the plasma membrane brings these two proteins in close proximity, allowing PDK1 to phosphorylate and activate Akt which in turn phosphorylates target proteins affecting cell growth, cell cycle progression, and cell survival [2]. The activation of the PI3K pathway is controlled by the 3′-phosphate lipid phosphatase PTEN (phosphatase and tensin homolog deleted on chromosome 10) which prevents the accumulation of PIP_3_ converting it back into PIP_2_ [3]. Members of class I PI3Ks are further divided into two subfamilies, class IA consisting of a p85α, p85β or p55γ regulatory subunit and a p110α, p110β or p110δ catalytic subunit, and class IB consisting of a p101 regulatory subunit and a p110γ catalytic subunit, which receive activation inputs from tyrosine kinases or GTPase signaling, respectively [1]. Unlike the ubiquitous p110α and p110β, p110δ and p110γ isoforms are preferentially expressed in leucocytes [4]. PI3K regulates many steps in the development, differentiation, and activation of T-cells. p110δ and p110γ are widely involved in thymocyte development and differentiation, especially during β-selection and transition between immature DP (double positive) and mature SP (single positive) thymocytes [4, 5]. Nevertheless, PI3K subunits seem to have a redundant role, and other isoforms could markedly impact T cell development [6]. Due to the crucial role of PI3Ks in regulating cell cycle, metabolism, and survival, the PI3K signaling cascade is one of the most frequently altered pathways in human cancers [7–9], and different compounds targeting members of the PI3K network have been developed and entered clinical trials [1].

T-cell acute lymphoblastic leukemia (T-ALL) is an aggressive neoplastic disorder of T-lymphocytes characterized by a poor clinical outcome, especially for relapsed patients [10]. In T-ALL, the PI3K pathway is frequently up-regulated mainly due to alterations, including phosphorylation, oxidation and gene deletion/mutation, that affect PTEN function [11–13]. Deregulation of the PI3K signaling pathway confers a proliferative advantage to malignant cells and might contribute to drug-resistance mechanisms. Therefore, targeting PI3K pathway may be an attractive novel therapeutic intervention in T-ALL. However, which class of agents among isoform-specific or pan-inhibitors can achieve the greater efficacy is still an open question. Indeed, if on the one hand the use of selective inhibitors might reduce systemic toxicity, on the other hand pan-inhibitors could display increased efficacy. In relation to this issue, it has been documented that PTEN-null T-ALL cells exclusively relied on p110γ and p110δ, as their combined absence decreased the tumor incidence in a PTEN-deficient mouse model, suggesting their predominant roles in sustaining malignant transformation [14]. Moreover, in human T-ALL cells devoid of PTEN, pharmacological blockade of both p110γ and p110δ impacted on tumor cell proliferation and survival, supporting the relevance of these isoforms as therapeutic targets for T-ALL treatment [14]. On the contrary, a more recent study highlighted the importance of blocking all class I PI3K isoforms to efficiently inhibit cell proliferation in PTEN deficient T-ALL cell lines [15]. However, in T-ALL patients *PTEN* genomic alterations are low frequency events, as *PTEN* gene deletions and mutations predicted to cause protein truncation occur collectively in about 10% of T-ALL cases [11, 12, 16]. In T-ALL, the predominant mechanisms responsible for PTEN functional inactivation and constitutive PI3K pathway activation are phosphorylation and/or oxidation, which have been detected at level above of control thymocytes in 91.7% and 81.3% of primary T-ALL samples, respectively [11]. Therefore, in the present study we aimed to further investigate the effects of PI3K inhibition in both *PTEN* deleted and non deleted T-ALL cell lines. For this purpose, we employed a pharmacological approach to compare the effects of selective and PI3K pan-inhibition. We used compounds which specifically target p110α, p110β, p110γ, and p110δ PI3K catalytic subunits, along with dual p110γ/p110δ and pan-PI3K inhibitors, and we evaluated their effects on leukemic cell proliferation and survival. Our results demonstrated that PI3K pan-inhibition exerted the most powerful effects on leukemic cell proliferation and survival in all the tested cell lines, irrespectively of *PTEN* status, with the possible exception of Loucy cells. Therefore, our findings strongly support clinical application of class I PI3K pan-inhibitors rather than dual γ/δ or single-isoform inhibitors for the treatment of the major part of T-ALL patients.

## RESULTS

### *In vitro* assessment of PI3K inhibitor effects on cell viability

In order to establish the role of the different PI3K catalytic subunits in supporting leukemic cells proliferation and survival, we exploited a pharmacological approach by using selective inhibitors, dual p110γ/δ, or pan-inhibitors. The pan-inhibitor BKM-120 has been evaluated in both preclinical hematologic and solid tumor models [17, 18] and phase I clinical trials [19–21], whereas ZSTK-474 [22–24] and PIK-90 [15] efficacy has been assessed only in preclinical models. To specifically inhibit p110α, p110β, p110δ, and p110γ we employed A-66, TGX-221, CAL-101, and AS-605240, respectively, whose selectivity has been reported elsewhere [14, 15, 25], and that, at least in several instances, have shown effectiveness in hematological malignancies [26]. Because of the prominent role of p110δ and p110γ isoforms in T-lymphocytes [5], effects of the γ/δ dual inhibitor IPI-145, as well as of a combination consisting of CAL-101 and AS-605240 were also evaluated. Several clinical trials have shown the efficacy of CAL-101, which displayed substantial anti-leukemic effects as single agent in both chronic lymphocytic leukemia (CLL) [27] and indolent non-Hodgkin lymphoma (iNHL) [28] patients with an acceptable safety profile. On this basis, the dual inhibitor IPI-145, initially developed as an anti-inflammatory drug [29], has been tested in phase I clinical trials enrolling relapsed/refractory lymphoma [30] or advanced CLL [31]. Results suggested that the drug is safe and effective and encouraged further evaluation of IPI-145 as a targeted drug also in newly diagnosed CLL patients.

**Figure 1:**
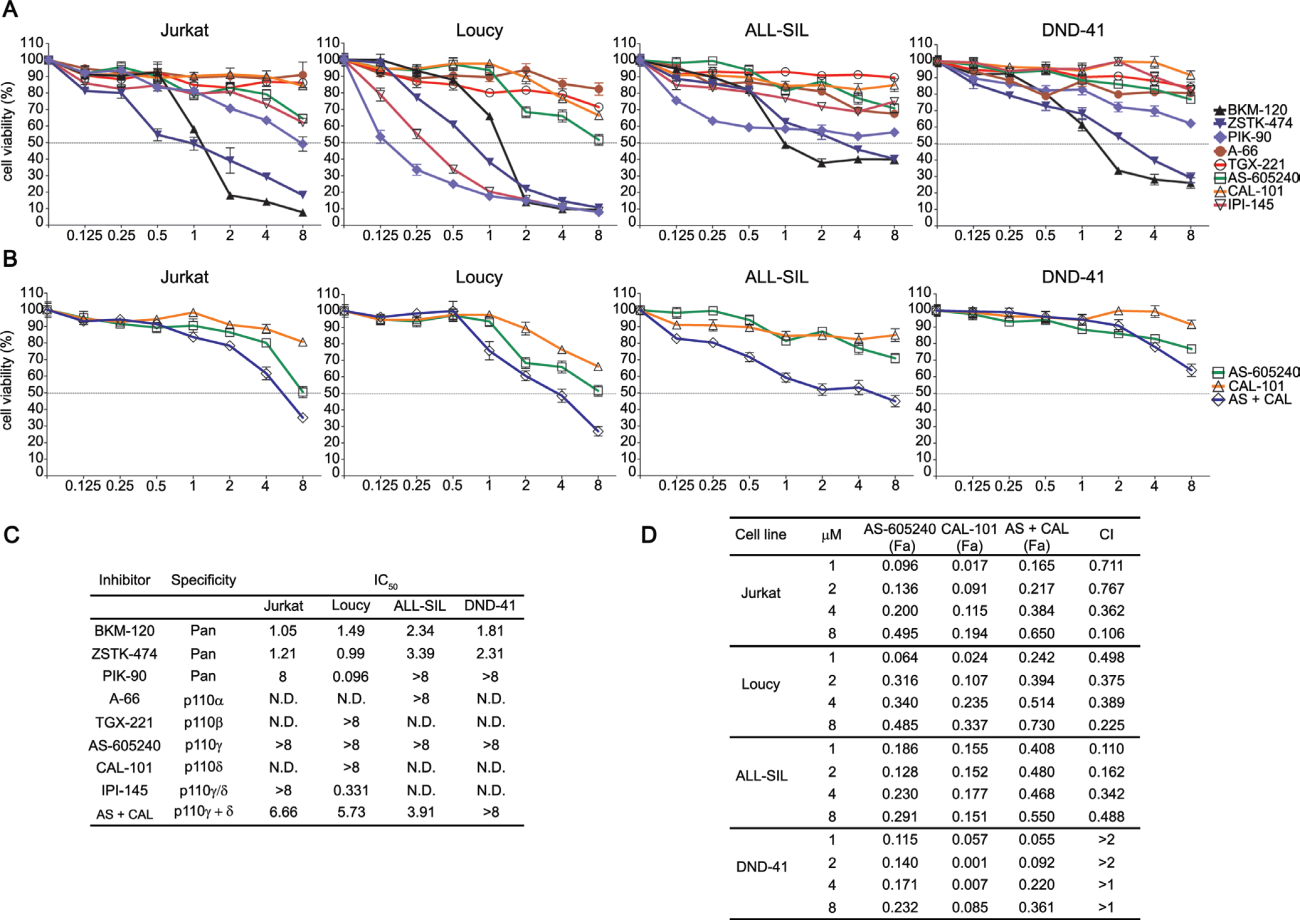
Inhibition of all the PI3K catalytic isoforms exerts the strongest effects on T-ALL cell line viability **(A and B)** MTT analysis of *PTEN* deleted (Jurkat, Loucy) and non deleted (ALL-SIL, DND-41) T-ALL cell lines treated for 48 h with increasing concentration of PI3K inhibitors (BKM-120, ZSTK-474, and PIK-90 are pan-inhibitors; A-66, TGX-221, AS-605240, CAL-101 are selective inhibitors of p110α, p110β, p110γ, and p110δ, respectively; IPI-145 is a dual inhibitor of p110γ/δ). (B) Effects of the combination consisting of AS-605240 and CAL-101 on cell viability. **(C)** IC_50_ values obtained through MTT assays after 48 h treatment with increasing concentrations of PI3K inhibitors. Three replicates per tested concentration and at least two independent experiments were performed (bars, SD). **(D)** Analysis of the effects of the AS-605240 (p110γ inhibitor) and CAL-101 (p110δ inhibitor) combination, which resulted synergistic in Jurkat, Loucy, and ALL-SIL cells (CIs 0.1–0.9). In DND-41 cells, CIs values > 2 indicate an antagonistic effect, whereas CIs > 1 are additive. (CI: combination index; Fa: Fraction affected).

Cells were cultured with increasing concentrations of the drugs for 48 h followed by metabolic activity assessment by MTT assay (Fig. 1A and 1C). In both *PTEN* deleted (Jurkat and Loucy) and *PTEN* non deleted (DND-41 and ALL-SIL) cells, growth rate decreased after treatment with BKM-120 and ZSTK-474 with IC_50_ values ranging between 1.05–2.34 μM for BKM-120 and 0.99–3.39 μM for ZSTK-474. Conversely, PIK-90 only mildly affected T-ALL cell line viability, with the exception of Loucy cells (IC_50_ 0.096 μM). As expected, selective inhibition of p110α, p110β, p110γ, and p110δ isoforms resulted ineffective, with IC_50_ values not attained at the tested concentrations. We further investigated the effectiveness of combining p110δ and p110γ inhibitors, by treating T-ALL cell lines with CAL-101 and AS-605240 at a fixed ratio (1:1). As shown in Fig. 1B and 1D, the inhibitors resulted in a strong (CI < 0.3) to moderate (CI < 0.9) synergism in ALL-SIL, Loucy, and Jurkat cells at concentrations above 1 μM, whereas in DND-41 cells the drug combination did not exert a synergistic but rather an antagonistic (at 1 and 2 μM) or additive (at 4 and 8 μM) effect. Nevertheless, IC_50_ values achieved by the combined treatment were much higher compared to those of pan-inhibitors (Fig. 1C). Interestingly, the dual p110γ/δ inhibitor IPI-145 was effective only in Loucy cells. Overall, PI3K isoform pan-inhibition was much more efficient in affecting T-ALL cell viability when compared to specific as well as dual p110γ/δ inhibition. Based on these results, we selected ZSTK-474 as pan-inhibitor and we used the concentration of 5 μM for the following experiments to simplify comparison of the results obtained with the other inhibitors.

### PI3K pan-inhibition affects cell proliferation in a PTEN independent fashion

**Figure 2:**
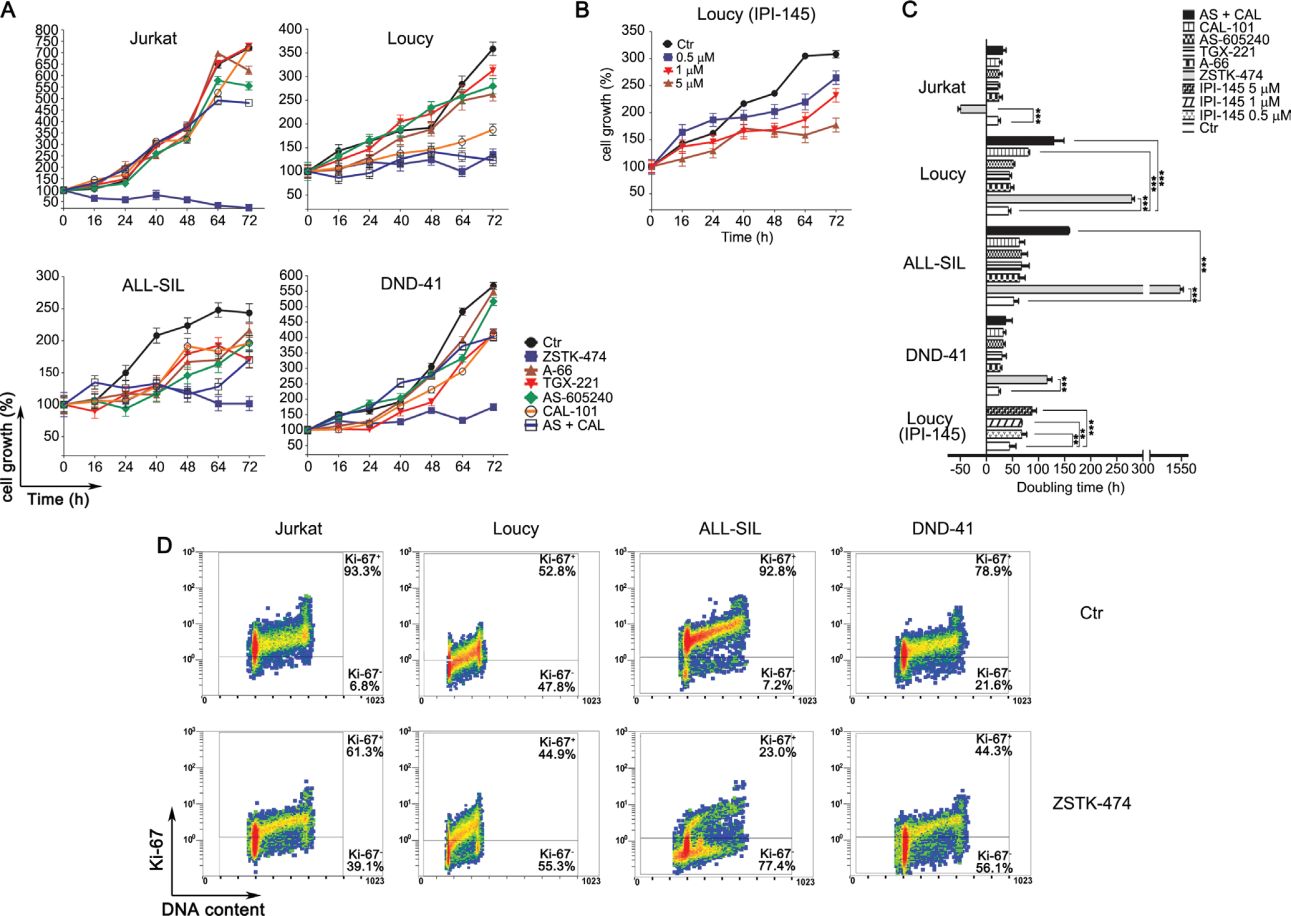
PI3K pan-inhibition impairs proliferation in T-ALL cell lines Growth curves of T-ALL cell lines treated with 5 μM of PI3K selective and pan-inhibitors **(A)** or with increasing concentration (0.5, 1 and 5 μM) of the dual inhibitor IPI-145 **(B)**. Viable cells were counted before treatment (0 h), and after 16, 24, 40, 48, 64 and 72 h of treatment. Cell growth was calculated as the percentage of viable cells compared to that at T 0 h. Four independent counts for each time point and two independent experiments for each cell line were performed (bars, SD). **(C)** Doubling time obtained from the cell count analysis. Increase in doubling time indicates a proliferation impairment. The negative doubling time observed in Jurkat cells indicates cell death induction. Asterisks indicate statistically significant differences with respect to untreated cells (*p < 0.05; **p < 0.01; ***p < 0.001). **(D)** Flow cytrometric analysis of the proliferation marker Ki-67. Cells were treated with 5 μM of the pan inhibitor ZSTK-474 for 72 h. Upper panel: control cells (untreated). Lower panels: treated cells.

We investigated in more detail the effects of the PI3K pathway inhibition on cell proliferation, by analyzing the long-term cell growth over 3 days post-treatment with the drugs. The pan-inhibitor ZSTK-474 significantly impaired cell proliferation in all the cell lines, independently from *PTEN* status, whereas p110α and p110β inhibition produced negligible effects (Fig. 2A). Specific and dual inhibition of p110γ and p110δ isoforms displayed an irregular pattern. Jurkat and DND-41 cell proliferation was unaffected, conversely in Loucy and ALL-SIL cells either p110δ inhibition or dual p110γ/δ inhibition significantly impaired cell growth (Fig. 2A). Compared to untreated controls, ZSTK-474 markedly slowed down the doubling time in Loucy, DND-41, and ALL-SIL cells, whereas a negative doubling time was estimated in Jurkat cells, suggesting cell death induction (Fig. 2C). Importantly, in Loucy cells, the only cell line responsive to IPI-145, proliferation was impaired already at 0.5 μM after treatment with this dual inhibitor (Fig. 2B and 2C).

To ascertain whether the observed effects of ZSTK-474 on cell growth rate were due to a proliferative impairment, we also evaluated by flow cytometry the expression of the proliferation marker Ki-67, a nuclear antigen which is a well established marker of cell proliferation [32]. In agreement with the growth analyses, Ki-67 decreased broadly in all the cell lines after 72 h treatment with the pan-inhibitor ZSTK-474 (Fig. 2D). These results demonstrated that overall PI3K pan-inhibition impaired cell proliferation more efficiently than dual p110γ/δ inhibition.

### Anti-proliferative effects of PI3K inhibitors are independent from total PIP_3_ level reduction

**Figure 3:**
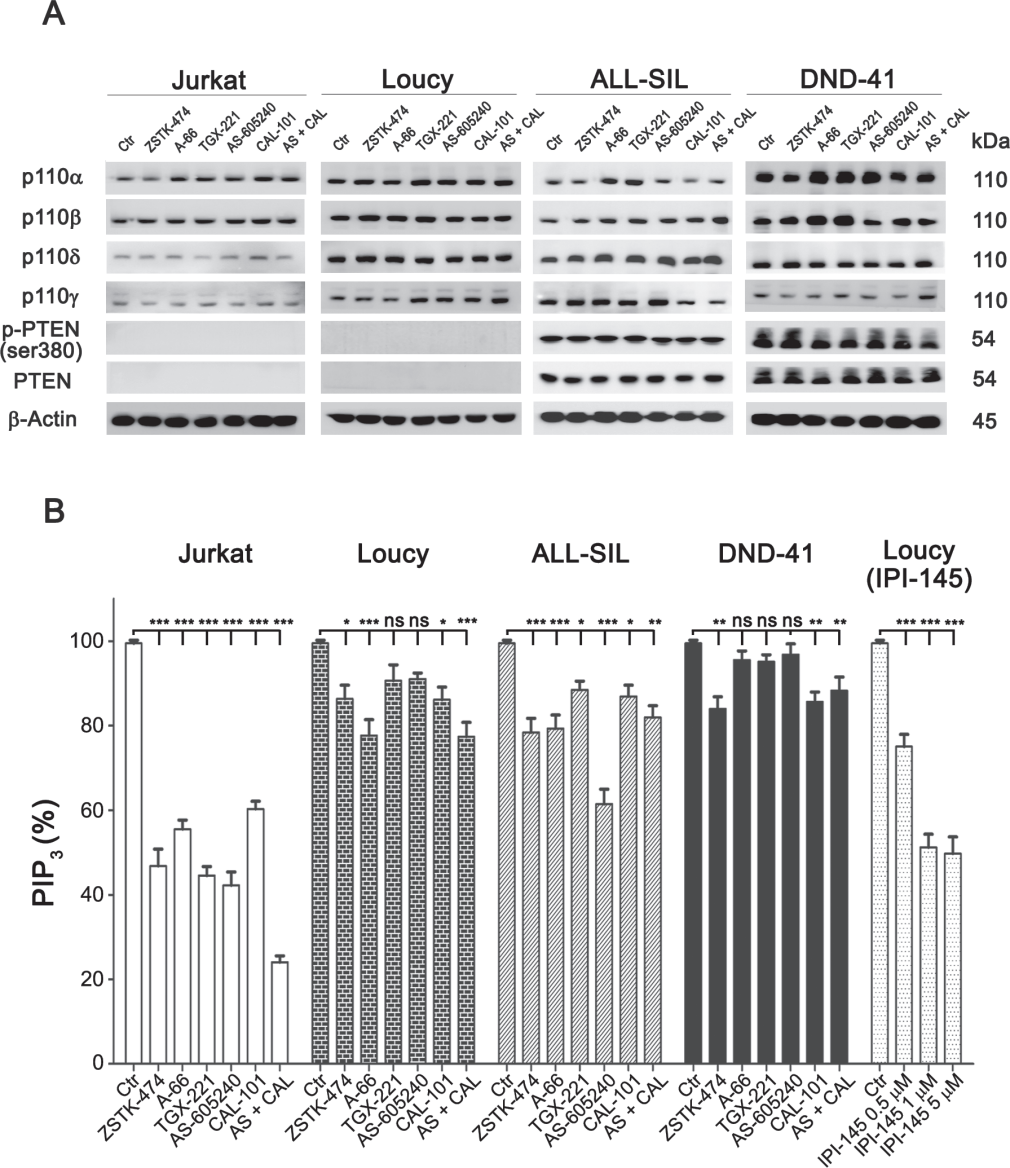
Expression of PI3K catalytic subunits and PTEN in T-ALL cell lines, and effects of PI3K inhibition on total PIP_3_ levels **(A)** Western blot analysis. Cells were cultured for 6 h with the different inhibitors, as indicated, and western blot analysis was then performed. *PTEN* non deleted cell lines express abundantly PTEN protein, however PTEN is phosphorylated at Ser380, which is an inhibitory site of its lipid phosphatase activity. **(B)** Flow cytomety quantification of the second messenger PIP_3_ in cells treated with 5 μM of PI3K inhibitors for 6 h. Bars, SD. Asterisks indicate statistically significant differences with respect to untreated cells (*p < 0.05; **p < 0.01; ***p < 0.001).

Firstly, we investigated cell lines with regard to PI3K isoforms and PTEN expression. As shown in Fig. 3A, the catalytic subunits p110α, −β, −γ and −δ were expressed in control as well as treated samples to a similar extent. As expected, PTEN protein was absent in Jurkat and Loucy cells, but abundantly expressed and unaffected by drug treatments in ALL-SIL and DND-41 cells. Nevertheless, in both of these cell lines, PTEN was phosphorylated at Ser380, which is a marker of PTEN posttranslational inactivation by CK2 and consequent PI3K pathway activation [12, 33].

To evaluate the impact of the inhibitors on PI3K activity, total PIP_3_ levels were then quantified by flow cytometry [11]. Pan-inhibition was able to induce a significant reduction of PIP_3_ in all cell lines after a 6 h treatment with the drugs. Regarding the isoform selective inhibitors, all the compounds induced a decrease in PIP_3_ levels, suggesting that each PI3K catalytic isoform could contribute to PIP_2_ phosphorylation in T-ALL cells (Fig. 3B). However, there were differences related to the cell type. While in Jurkat cells all the inhibitors drastically decreased PIP_3_, in the other cell lines a significant decrease in PIP_3_ could be detected only with some of the inhibitors (CAL-101 in DND-41; A-66, AS-605240 and combination of AS-605240 plus CAL-101 in ALL-SIL; A-66, CAL-101 and combination of AS-605240 plus CAL-101 in Loucy cells). Moreover, PIP_3_ decreased in a concentration-dependent fashion in Loucy cells treated with the dual p110γ/δ inhibitor IPI-145 (0.5, 1 and 5 μM; Fig. 3B). These observations suggested a peculiar addiction to PI3K isoforms of the different cell lines with regard to PIP_2_ phosphorylation, suggesting a potential influence of cellular-specific mechanisms in PIP_3_ generation. However, PIP_3_ reduction did not fully correlate with the observed anti-proliferative effects induced by the inhibitors.

Taken together, these data demonstrated that each isoform can sustain PIP_3_ synthesis in T-ALL cells, but PIP_3_ total cellular amount was not fully related to cellular proliferation.

### PI3K pan-inhibition impairs Akt-mediated signaling

**Figure 4:**
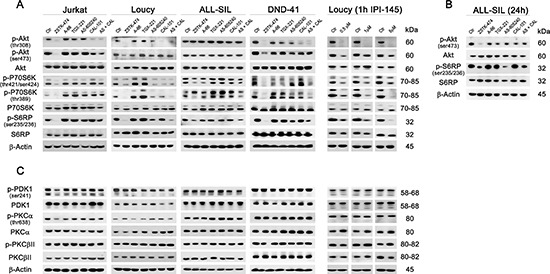
PI3K pan-inhibition impacts on the PI3K/Akt/mTOR pathway Cells were cultured for 6 h **(A and C)** or 24 h **(B)** in the presence of 5 μM of the different PI3K inhibitors, as indicated, and western blot analysis was then performed. The pan-inhibitor ZSTK-474 and the combination of p110γ and p110δ inhibitors (AS-605240 and CAL-101) induced the dephosphorylation of the main PI3K downstream targets Akt, P70S6K, and S6RP (A and B) but only ZSTK-474 decreased both the phosphorylated residues of Akt, Thr308 and Ser473. Only in Loucy cells, the dual p110γ/δ inhibitor IPI-145 exerted a concentration-dependent activity on PI3K downstream targets. (C) The phosphorylation of PDK1, PKCα and PKCβII was not modulated by inhibition of PI3K. Thirty μg of protein was blotted to each lane. Antibody to β-Actin served as a loading control. Molecular weights are indicated at right. Ctr, untreated cells.

We next examined the effects of the different inhibitors on signaling downstream of PI3K. One of the major PI3K targets is the serine/threonine kinase Akt, which is recruited to the plasma membrane through direct interaction with PIP_3_ and subsequent phosphorylation on Thr308 by PDK1 and Ser473 by mTORC2 for full activation [7]. In all cell lines, selective p110α, −β or −γ inhibition was unable to reduce Akt phosphorylation at Thr308, whereas both p110δ and dual p110γ/δ inhibition induced a comparable decrease, suggesting a major role for the p110δ isoform in the phosphorylation of this Akt amino acidic residue (Fig. 4A). Conversely, pan-inhibition exerted the strongest effect on Akt activation, with a complete abrogation of phosphorylation at Thr308. Most importantly, only pan-inhibition induced a significant reduction of Akt phosphorylated at Ser473, either after 6 h (Jurkat, Loucy, and DND-41 cells; Fig. 4A) or 24 h (ALL-SIL cells; Fig. 4B) treatment, suggesting mTORC2 inhibition. Analysis of Akt downstream targets showed a congruent pattern of dephosphorylation, with a decrease in p-P70S6K both at Thr421/Ser424 (auto-inhibitory domain) and at Thr389 (mTORC1 phosphorylation site), and p-S6RP at Ser235/236, after pan- or, to a lesser extent, dual p110γ/δ inhibition (Fig. 4A). In all cases, total protein levels were unaffected. Consistently with the cell viability and proliferation analyses, only in the Loucy cell line down-modulation of p110γ/δ activity with the dual inhibitor IPI-145 was as effective as PI3K pan-inhibition and displayed a concentration-dependent trend (Fig. 4A).

To assess if other targets beyond Akt could be affected by PI3K inhibition, we further investigated PDK1 and some of its downstream targets, PKCα and PKCβ (Fig. 4C). We observed that neither selective, nor dual or pan-inhibitors reduced phosphorylated levels of PKC isoforms [34]. Therefore, block of PI3K activity mainly inhibited Akt and its downstream targets.

### Anti-proliferative activity of PI3K pan-inhibition acts through cell cycle arrest and caspase-independent apoptosis

**Figure 5:**
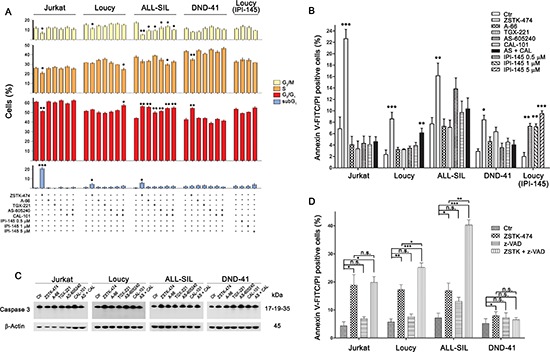
PI3K pan-inhibition affects cell cycle progression and induces caspase-independent cell death **(A)** Flow cytometry analysis of PI-stained cells treated with 5 μM of the different PI3K inhibitors (as indicated) for 48 h. The pan-inhibitor ZSTK-474 increased the subG_1_ and/or the G_0_/G_1_ cell fraction, with the consequent decrease of the other cell cycle phases, whereas the dual p110γ/δ exerted limited effects on the cell cycle progression. The dual inhibitor IPI-145 did not perturb the cell cycle of the Loucy cell line. **(B)** Flow cytometric analysis of Annexin V-FITC/PI–stained T-ALL cells treated with 5 μM of the different PI3K inhibitors for 48 h documented a significant increase in the cell death fraction with respect to untreated cells only after PI3K pan-inhibition. However, the dual inhibitor IPI-145 exerted a concentration-dependent pro-apoptotic effect on Loucy cells. **(C)** Western blotting documented that PI3K inhibition did not induce caspase-3 activation. Thirty μg of protein was blotted to each lane. Antibody to β-Actin served as a loading control. Molecular weights are indicated at right. **(D)** T-ALL cells were treated with 5 μM ZSTK-474 for 48 h with or without the pan-caspase inhibitor z-VAD-fmk (50 μM) and the cell death fraction was assessed using Annexin V-FITC/PI staining. Caspase inhibition did not reduce cytotoxicity. Results are the mean of three different experiments ± SD. Asterisks indicate statistically significant differences with respect to untreated cells (*p < 0.05; **p < 0.01; ***p < 0.001).

Since PI3K/Akt signaling controls different cellular pathways, we examined the inhibitor effects on cell cycle and induction of apoptosis. Interestingly, cell cycle was affected in all the cell lines following pan-inhibition (ZSTK-474 treatment for 48 h) (Fig. 5A). Flow cytometric analysis documented an accumulation of cells in the G_0_/G_1_ phase of the cell cycle and a consequent decrease of cells in the S or G_2_/M phases, as previously reported [23], which reached a statistical significance in ALL-SIL and DND-41 cells. Moreover, in Jurkat, Loucy, and ALL-SIL cells, ZSTK-474 increased the subG_1_ cell fraction, which comprises death cells. Less dramatic effects were observed with the other inhibitors, which affected only ALL-SIL and Loucy cells. In particular, the combination of AS-605240 and CAL-101 altered cell cycle phase distribution in Loucy cells, by increasing the G_0_/G_1_ fraction. However, the dual γ/δ inhibitor IPI-145 had no effects.

Annexin V-FITC/PI analysis confirmed a significant increase in apoptotic cells following treatment with ZSTK-474 for 48 h in all the cell lines, whereas dual p110γ/δ inhibition had limited effects, inducing a significant cell death only in the Loucy cell line (Fig. 5B).

Moreover, western blot analysis demonstrated the absence of cleaved effector caspase 3 both at 24 and 48 h of treatment with the inhibitors (Fig. 5C and data not shown) suggesting that caspases did not contribute to cell death. To formally prove that, we treated T-ALL cell lines with ZSTK-474 in the presence or absence of the pan-caspase inhibitor N-benzyloxycarbonyl-Val-Ala-Asp-fluoromethylketone (z-VAD-fmk). As presented in Fig. 5D, cell death induced by pan-inhibition was not affected by caspase inhibition.

Overall, these results demonstrated that PI3K inhibition caused cell cycle arrest in G_0_/G_1_ cell phase but only pan-inhibition was able to efficiently induce cell death with a caspase-independent mechanism.

### Autophagy is a protective mechanism against PI3K pan-inhibition

**Figure 6:**
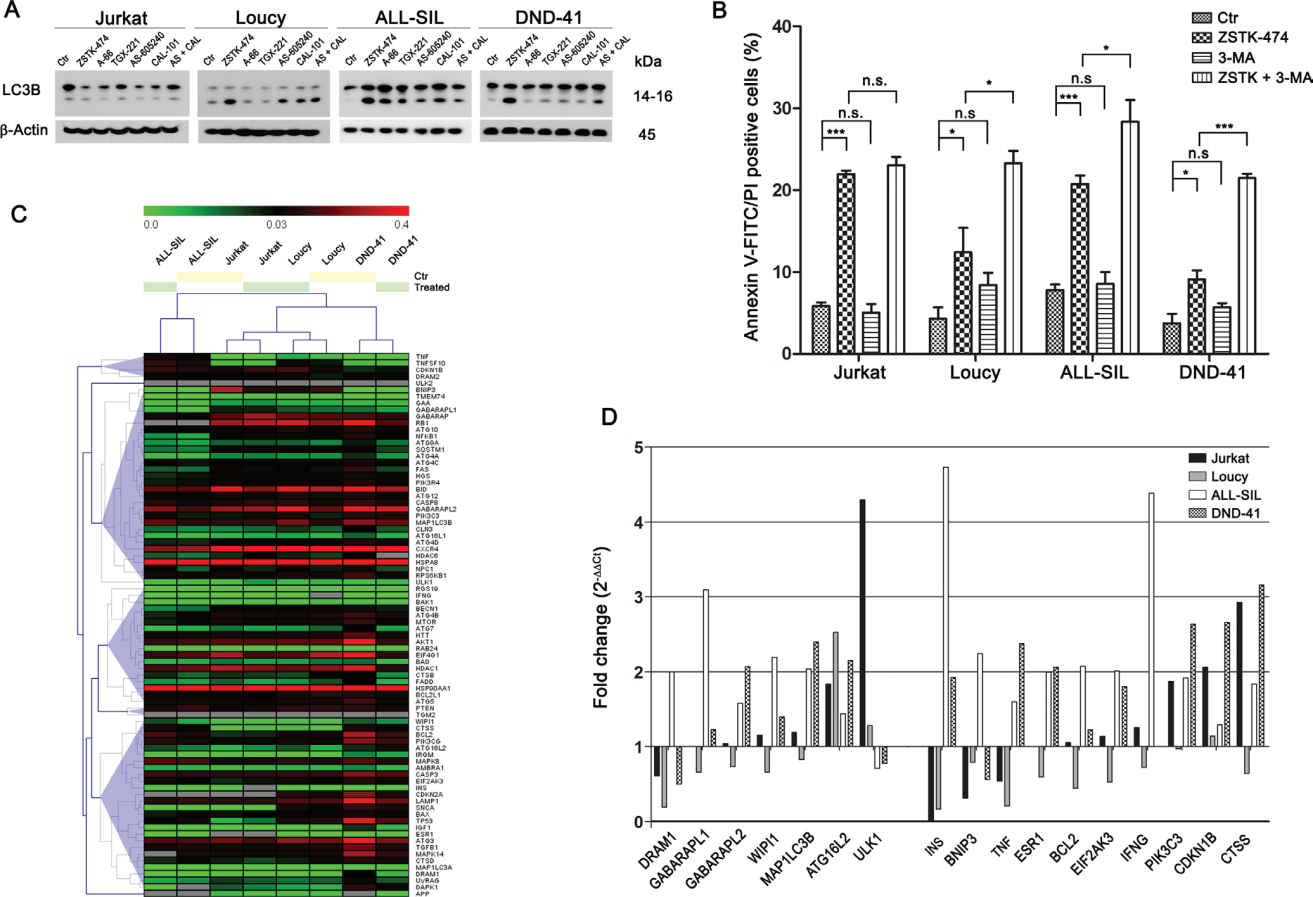
PI3K pan-inhibition induces autophagy which plays a protective role **(A)** Western blotting demonstrated autophagy activation in Loucy, ALL-SIL, and DND-41 cell lines in response to PI3K inhibition. Thirty μg of protein was blotted to each lane. Antibody to β-Actin served as a loading control. Molecular weights are indicated at right. **(B)** T-ALL cells were treated with 5 μM ZSTK-474 for 48 h with or without the autophagy inhibitor 3-MA (200 μM) and cell death fraction was assessed by Annexin V-FITC/PI staining. Autophagy inhibition significantly increased cell death in Loucy, ALL-SIL, and DND-41 cell lines, whereas it did not affect Jurkat cells. Results are the mean of three different experiments ± SD. Asterisks indicate statistically significant differences with respect to untreated cells (*p < 0.05; **p < 0.01; ***p < 0.001). **(C)** Real-time PCR expression profiling of 82 autophagy-related genes in T-ALL cell lines untreated (Ctr) or treated for 24 h with 5 μM ZSTK-474 were visualized using an unsupervised heat map. Data are presented as 2^−ΔCt^ (ΔCt = Ct _target gene_ – Ct _RLP0_). **(D)** Histograms represent the relative gene expression of several autophagy-related genes in T-ALL cells treated with ZSTK-474 and compared to untreated paired sample. Data are presented as 2^−ΔΔCt^ (ΔΔCt = ΔCt _treated sample_ – ΔCt _Ctr sample_). When fold change values are = 1, the regulation in treated samples is equal to the paired control sample. When fold change values are > 1 or < 1, the autophagy-related genes are up- or down-regulated, respectively, compared to untreated samples.

Autophagy is a homeostatic cellular process which regulates protein and organelle turnover through their lysosomal destruction [35]. However, autophagy also executes cell death, and autophagic cell death is one of the better recognized caspase-independent programmed cell death mechanisms [36]. To analyze possible autophagy induction, we investigated the expression of LC3B I/II, a recognized autophagy marker [37]. Western blot analysis demonstrated a marked increase in LC3B II, the lipidated form of the protein which is bound to the autophagosome membranes, in Loucy, ALL-SIL and DND-41 cells, especially following 24 h ZSTK-474 treatment, whereas no changes were observed in Jurkat cells (Fig. 6A). Because PI3K pan-inhibition with ZTK-474 treatment induced a considerable percentage of cell death in Jurkat cells compared to the other cell lines, we supposed a protective role of autophagy in this context. To test our hypothesis, we inhibited autophagy with the early-stage autophagy inhibitor 3-methyladenine (3-MA) and subsequently evaluated cell death induced by treatment with ZSTK-474. The results demonstrated that 3-MA increased the cytotoxic effect of pan PI3K inhibition, as the percentage of Annexin V/PI positive cells was significantly higher in Loucy, ALL-SIL, and DND-41 cells compared to that of samples treated with ZSTK-474 alone (Fig. 6B). On the contrary, in Jurkat cells, where pan-inhibition did not induce LC3B lipidation, inhibition of autophagy did not increase cytotoxicity (Fig. 6B). To ascertain whether the different behavior between Jurkat cells and the other cell lines was related to a different modulation of autophagy-related genes, a screening for gene expression was performed using a quantitative real-time PCR assay which interrogates 82 genes related to the autophagic pathway (Tab. 1 and Fig. 6C). Unsupervised hierarchical clustering showed similarities in autophagy gene expression in each paired cell line (untreated and treated samples) (Fig. 6C), although untreated Loucy cells showed a basal higher expression of these genes compared to the other cell lines. Moreover, no differentially clustered transcripts were observed in Jurkat cells, despite the fact that this cell line did not activate the autophagy process following PI3K pan-inhibition. Nevertheless, we further investigated the modulation of autophagy in more detail, by comparing for each cell line untreated and treated samples and assessing for each gene the fold change, expressed as 2^−ΔΔCt^. A 24 h treatment with ZSTK-474 had limited effects on autophagy at a transcriptional level, as the majority of genes resulted expressed equally to the control (2^−ΔΔCt^ = 1) or slightly reduced (2^−ΔΔCt^ < 1), especially in Loucy cells (Fig. 6C). Nevertheless, in some instances we observed *a* > 2 fold increase both in components of the autophagic machinery and in genes involved in autophagy regulation (Tab. 2 and Fig. 6D). In particular, in Loucy, ALL-SIL, and DND-41 cells, PI3K pan-inhibition increased the expression of *DRAM1*, *GABARAPL1*, *GABARAPL2*, *WIPI1*, *MAP1LC3B*, and *ATG16L2*, all involved in autophagic vesicle nucleation and expansion, as well as increased expression of genes involved in autophagy induction and regulation (*INS*, *PIK3C*) or prosurvival genes (*BCL2*, *EIF2AK3*). In contrast, in Jurkat cells, pan-inhibition had limited effects on autophagy-related gene induction, as the only upregulated gene was *ULK1*. However, we observed *a* > 2 fold change in expression of the tumor suppressor gene *CDKN1B*, as well as in *CTSS* (cathepsin S) gene. Interestingly, low levels of cathepsin S or its pharmacological inhibition have been related to the induction of autophagy in cancer cells [38, 39].

**Table 1:** Autophagy-related genes analyzed using real-time PCR microarrays

**Autophagy Machinery Components**
*AMBRA1, ATG10, ATG12, ATG16L1, ATG16L2, ATG3, ATG4A, ATG4B, ATG4C, ATG4D, ATG5, ATG7, ATG9A, BECN1, DRAM1, GABARAP, GABARAPL1, GABARAPL2, IRGM, LAMP1, MAP1LC3A, MAP1LC3B, NPC1, RAB24, RGS19, ULK1, ULK2, WIPI1*
**Regulation of Autophagy**
*AKT1, APP, BAD, BAK1, BAX, BCL2, BCL2L1, BID, BNIP3, CASP3, CASP8, CDKN1B, CDKN2A, CLN3, CTSB, CTSD, CTSS, CXCR4, DAPK1, DRAM2, EIF2AK3, EIF4G1, ESR1, FADD, FAS, GAA, HDAC1, HDAC6, HGS, HSPA8, HSP90AA1, HTT, IFNG, IGF1, INS, MAPK14, MAPK8, MTOR, NFKB1, PIK3CG, PIK3C3, PIK3R4, PTEN, RB1, RPS6KB1, SNCA, SQSTM1, TGFB1, TGM2, TMEM74, TNF, TNFSF10, TP53, UVRAG*

**Table 2:** Autophagy-related genes up-regulated after PI3K pan-inhibition

**Autophagy Machinery Components**		
***DRAM1***	DNA-damage regulated autophagy modulator 1	Lysosomal modulator of autophagy induced by p53
***GABARAPL1***	GABA(A) receptor-associated protein like 1	essential for autophagosome maturation
***GABARAPL2***	GABA(A) receptor-associated protein-like 2	essential for autophagosome maturation
***MAP1LC3B***	Microtubule-associated protein 1 light chain 3 beta	involved in formation of autophagosomes
***ATG16L2***	Autophagy-related 16-like 2	May play a role in autophagy during membrane biogenesis
***ULK1***	unc-51 like autophagy activating kinase 1	Regulate the formation of autophagophores (upstream *PIK3C*)
***WIPI1***	WD repeat domain, phosphoinositide interacting 1	Required for autophagosome formation (downstream *ULK1* and *PIK3C*)
**Regulation of Autophagy**		
***INS***	Insulin	Peptide hormone
***BNIP3***	BCL2/adenovirus E1B 19kDa interacting protein 3	May positively modulate autophagydisplacing Bcl-2 from the Bcl-2/Beclin 1 complex
***TNF***	Tumor necrosis factor	Cytokine
***ESR1***	Estrogen receptor 1	Regulate autophagy core proteins
***BCL2***	B-cell CLL/lymphoma 2	Promote cellular survival
***EIF2AK3***	Eukaryotic translation initiation factor 2-alpha kinase 3	Repress global protein synthesis. Critical effector of unfolded protein response (UPR)
***IFNG***	Interferon gamma	Cytokine
***PIK3C3***	Phosphatidylinositol 3-kinase, catalytic subunit type 3	Involved in initiation and maturation of autophagosomes
***CDKN1B***	Cyclin-dependent kinase inhibitor 1B (p27Kip1)	Cell cycle regulator. Its degradation is required for G_1_ cell phase progression
***TP53***	Tumor protein p53	Tumor suppressor
***CTSS***	Cathepsin S	Cysteine lysosomal protease involved in autophagic flux regulation

These results demonstrated that PI3K pan-inhibition could induce autophagy which plays a protective role in T-ALL cells, and suggested that autophagy activation in the different T-ALL cell lines might be dependent on a different gene expression regulation.

## DISCUSSION

PI3K signaling is often deregulated in malignancies and contribute to the oncogenic process. The mechanisms responsible for class I PI3Ks up-regulation diverge among the distinct tumors. For example, gain of function mutations in *PIK3CA*, that encodes the catalytic subunit p110α, have been detected in a wide variety of human solid cancers [40], whereas p110δ is implicated in cancers derived from B lymphoid cells [41, 42]. Recently, the oncogenic potential of p110β [43] has emerged in breast [44] and prostate cancers [45]. Tumor cells addiction to the activity of specific class I PI3K isoforms had led to the development of therapies based on the application of selective PI3K inhibitors which target the catalytic subunits [46]. In T-ALL, PI3K signaling up-regulation has been found in nearly 90% of cases [11] and targeting PI3K is an attractive novel strategy to treat these patients. Nevertheless, at present is still controversial the role carried out by the different PI3K catalytic subunits in T-ALL and, therefore, which might be the most useful therapeutic strategy. It is well established that both p110γ and p110δ, enriched in leucocytes, are involved in thymocyte development [5]. The catalytic subunits p110α and p110β are ubiquitously expressed so that it is extremely difficult to dissect their role in lymphocytes, due to the embryonic lethality induced by loss of either p110α or p110β [47]. However, there is a complex interplay between the class I PI3K members, as inhibition or loss of a specific isoform might be compensated by the others in interleukin-3-dependent mouse hematopoietic cells [48]. Moreover, recent findings have highlighted that in solid tumors inhibition of a single PI3K isoform (either α or β) could be compensated by reactivation of another isoform [49, 50].

Recently, Subramanian et al., by employing the dual γ/δ inhibitor CAL-130, proposed the predominant role of p110γ and p110δ in *PTEN* deleted T-ALL, suggesting the possibility to target this malignancy by inhibiting specifically these isoforms [14]. It should be underscored, however, that after this initial report, no other papers dealing with the effects of CAL-130 have been published. In contrast, a more recent work contradicted these results, showing higher cytotoxic effects of the PI3K pan-inhibitor, PIK-90, in *PTEN* deleted T-ALL cell lines [15]. However, both of these studies did not take into account that *PTEN* deletions or inactivating gene mutations are relatively rare in primary T-ALL patients, whereas posttranslational inactivation of PTEN is a much more frequent event [11]. In light of those contradictory findings, we have used isoform-selective, pan- and dual p110γ/δ inhibitors to compare their effects in both *PTEN* deleted and non deleted T-ALL cell lines. Our results demonstrated that blockage of all the class I PI3K catalytic isoforms exerted a greater anti-cancer effect compared to dual p110γ/δ inhibition, as exemplified by the lower IC_50_ attained in all the cell lines, whereas isoform-selective inhibition produced negligible effects. Among the three PI3K pan-inhibitors we tested, PIK-90 was effective only in Loucy cells, highlighting that chemical structure might affect drug efficacy. Moreover, only Loucy cells were sensitive to the dual γ/δ inhibitor, IPI-145. The peculiar sensitivity of Loucy cells to PI3K pathway inhibition is remarkable, as this cell line displays a transcriptional signature similar to that of early T-precursor (ETP)-ALL, a T-ALL subtype associated with an extremely poor prognosis [51]. Therefore, it will be very critical to test this drug on primary cells derived from patients with ETP-ALL.

Moreover, we did not observe differences between *PTEN* deleted and non deleted cell lines. Indeed, despite the expression of PTEN protein, we confirmed that in non deleted cells, PTEN was phosphorylated at Ser380 and thus inactivated. This observation underlines the importance of assessing PI3K pathway activation in T-ALL patients rather than just *PTEN* deletions/mutations, for a better evaluation of patient outcome or possible therapeutic intervention with pathway modulators. We also observed a decrease in total PIP_3_ levels following inhibition of each PI3K catalytic subunit, suggesting that all the PI3K isoforms contribute to its synthesis in T-ALL cells. However, inhibitor effects on PIP_3_ levels did not fully correlate with their cytotoxicity. This apparent contradiction might be due to the presence of different PIP_3_ pools which mediate specific cellular processes, so that reduction of total PIP_3_ could not fully reflect the impairment of cell proliferation/survival mechanisms [52]. Moreover, it is emerging that PI3K could control other downstream targets in cancer cells, including serum/glucocorticoid-regulated kinase 3 (SGK3), in a manner which is independent from PIP_3_, but dependent on phosphatidylinositol 3-phosphate [53].

Only PI3K pan-inhibition switched off Akt signaling, as demonstrated by the reduction or complete abrogation of both Thr308 and Ser473 p-Akt levels. It has been previously reported that a limited PI3K activity is sufficient to support cell survival and proliferation and, consequently, complete PI3K inhibition is required to induce cell death [48]. Consistent with that, in spite of changes in cell cycle progression, we observed a significant cell death induction almost exclusively after PI3K pan-inhibition, with the exception of Loucy cells where the dual inhibition of p110γ/δ was also effective. Importantly, we demonstrated that, at least in the case of ZSTK-474, the mechanisms involved in cell death are independent of caspase activity as a pan-caspase inhibitor did not reduce cell death.

Numerous studies have highlighted the existence of various types of programmed cell death besides apoptosis, including autophagy, an important catabolic mechanism which can play both a pro-survival or pro-death role [54]. Importantly, PI3K and autophagy pathways are tightly related, as mTORC1 suppress autophagy by inhibiting ULK1, and the transcription factor FoxO3, which is inhibited by active Akt, regulates a number of autophagy-related genes, including *LC3*, *GABARAPL1*, *BNIP3*, *PIK3C3*, and *ULK1* [35, 55, 56]. Our findings demonstrated that activation of autophagy can sustain cell survival after PI3K pan-inhibition. In fact, inhibitors that interfere with the autophagic flux increased the cytotoxic effects of ZSTK-474. However, autophagy activation took place only in some T-ALL cell lines, suggesting the influence of a more complex cellular background. To address this issue we examined the expression of autophagy-related genes. No specific expression profiles resulted related to autophagy activation or PI3K pan-inhibition and overall gene expression of paired samples was similar. Neverthelss, inhibition of PI3K specifically modulated a few genes that might trigger autophagy. While the Loucy cell line displayed a higher autophagy gene expression profile already at basal level, we observed the up-regulation of genes involved in the autophagic machinery and regulation in all the T-ALL cell lines we studied. Increase of these transcripts appeared to be cell type-dependent, as ALL-SIL, DND-41 and, partially, Loucy cells, which activated autophagy after PI3K pan-inhibition, up-regulated numerous genes involved in the formation of autophagosomes (*DRAM1, GABARAPL1, GABARAPL2, MAP1LC3B, ATG16L2, WIPI1, PIK3C3*), as well as anti-apoptotic genes (*BCL2*) and genes of the unfolded protein response signaling (*EIF2AK3*). Conversely, in Jurkat cells, which did not activate autophagy, gene modulation was affected to a much lower extent and preferentially involved anti-proliferative targets, as the cell cycle inhibitor *CDKN1B*, and the autophagy inhibitor *CTSS*. These observations suggest a role for the PI3K pathway in modulating at a transcriptional level the complex relationship between pro- and anti-survival signals and ultimately the balance between autophagy and apoptosis. Of course, more studies are needed to clarify these relationships.

In conclusion, we have demonstrated a higher efficacy of PI3K pan-inhibition in both PTEN deleted and non deleted T-ALL cell lines. Although dual inhibition of p110γ/δ PI3K isoforms could be less toxic and reduce side effects [25], its efficacy might be limited only to the subset of T-ALL patients with ETP-ALL. Moreover, our findings shed light about the protective role of autophagy in case of PI3K pan-inhibition, supporting the evaluation of combining autophagy inhibitors for increasing citotoxicity induced by PI3K inhibition. Further investigation will be necessary to discriminate the cellular contexts responsible for autophagy activation. Addressing this question will be critical to select which T-ALL patients may best benefit from a therapeutic strategy involving class I PI3K inhibition. Indeed, progress in better understanding the biology of different T-ALL subtypes should help the development of personalized therapy targeted at blocking multiple defective signaling pathways of leukemic cells [57].

## MATERIALS AND METHODS

### Cell lines and reagents

Human T-ALL cell lines Jurkat and Loucy (*PTEN* deleted), DND-41 and ALL-SIL (*PTEN* non deleted) were cultured in RPMI-1640 medium (Life Technologies Italia, Monza, Italy) supplemented with 10–20% fetal bovine serum (Life Technologies), 100 U/ml penicillin and 100 μg/ml streptomycin (Sigma-Aldrich, Saint Louis, MO, USA) at 37°C in a humidified atmosphere of 5% CO_2_. PI3K inhibitors BKM-120, PIK-90, ZSTK-474, A-66, TGX-221, AS-605240, CAL-101 and IPI-145, the caspase inhibitor z-VAD and the autophagy inhibitor 3-MA, were from Selleck Chemicals (Houston TX, USA).

### Cell viability assay and cell number count

To test the effects of PI3K inhibitors, T-ALL cell lines were cultured for 48 h in the presence of the vehicle (DMSO 0.1%) or increasing drug concentrations, and cell viability was determined using the MTT [3-(4, 5-Dimethylthythiazol-2-yl)-2, 5-diphenyltetrazolium bromide] cell proliferation kit (Roche Diagnostic, Basel, Switzerland), according to manufacturer's instructions. For drug-combination experiments, a combination index (CI) number was calculated using the CalcuSyn software (Cambridge, UK) based on the Chou and Talalay method [58]. CI values between 0.1–0.9 define different grades of synergism, values between 0.9–1.1 are additive, whereas values > 1.1 are antagonistic. Growth curves were generated by counting viable and non-viable cell numbers by the Trypan blue dye exclusion method. Cells were seeded in 6-well plates, treated with the inhibitors (5 μM) or the vehicle alone (DMSO 0.1%) and counted at regular intervals up to 72 h. Doubling time was calculated with Roth V. 2006 (http://www.doubling-time.com/compute.php).

### Flow cytometry analysis of Ki-67 and PIP_3_

In order to evaluate effects on proliferation, Ki-67 antigen expression during the different phases of cell cycle was evaluated. After 72 h treatment, cells were permeabilized with methanol/acetic acid (3:1) and incubated with a primary antibody to Ki-67 (Cell Signaling Technology, Danvers, MA, USA). Afterwards, samples were stained for 1 h with a FITC-conjugated secondary antibody (Beckman Coulter, Miami, FL, USA) followed by further 20 min incubation with Propidium Iodide (PI). To detect PIP_3_ levels, control (DMSO 0.1%) and treated (6 h) cells were fixed in 4% paraformaldehyde for 15 min, permeabilized in 0.4% Triton X-100 for 10 min, washed in PSB 1X with 1% BSA and incubated over night at 4°C with a FITC-conjugated anti-PIP_3_ antibody (Echelon Biosciences Inc., Salt Lake City, UT). Analyses were performed on an FC500 flow cytometer (Beckman Coulter) with the appropriate software (CXP, Beckman Coulter).

### Annexin V-FITC/PI staining and cell cycle analysis

Apoptosis and cell cycle analysis were performed as previously described [59]. T-ALL cell lines were treated for 48 h with the different compounds (5 μM) or the vehicle alone (DMSO 0.1%). Analyses were performed on an FC500 flow cytometer (Beckman Coulter) with the appropriate software (CXP).

### Western blot

Western blotting was performed by standard methods, as previously described [60]. Cells were lysed using the M-PER Mammalian Protein Extraction Reagent supplemented with the Protease and Phosphatase Inhibitor Cocktail (Thermo Fisher Scientific Inc., Rockford, IL, USA). The PI3K p110δ antibody was from Santa Cruz Biotechnology (Heidelberg, Germany). All other primary and secondary antibodies were bought from Cell Signaling Technology.

### Gene expression analysis

Total RNA was isolated either from control (DMSO 0.1%) and from 24 h ZSTK-474 treated cells using RNeasy Mini Kit (QIAGEN, Valencia, CA) according to the manufacturer's instructions. RNA concentration was determined by measuring the absorbance at 260 nm; for all samples, the OD 260/OD 280 absorbance ratio was of at least 2.0. 3.5 μg of total RNA were reverse-transcribed into cDNA using the iScript^TM^ Advanced cDNA Syntesis Kit (Bio-rad, Hercules, CA, USA). Gene expression of specific autophagy markers was measured using the PrimePCR^TM^ Assay for real-time (Bio-rad). For each sample, cDNA was mixed with 2x SsoAdvanced^TM^ universal supermix (25 ng cDNA/reaction) containing SYBR Green (Bio-rad) and aliquoted in equal volumes to each well of the real-time PCR arrays. The quantitative PCR reaction was performed using a 7300 Real-Time PCR system (Applied Biosystems, Foster City, CA, USA). The quantitative PCR thermal protocol consisted of 95°C for 2 minutes, followed by 40 cycles of 95°C for 5 seconds and 60°C for 30 seconds. *RLP0* was used as control gene and the relative gene expression among samples was calculated as 2^−ΔCt^ [61]. These data were then subjected to hierarchical clustering using the Spearman's rank correlation metric and the average-linkage method and heatmaps were generated using the data analysis tool TIGR Multiexperiment Viewer (http://www.tm4.org/) [62]. To examine the effects of pan PI3K inhibition in the different cell lines, gene expression of the treated cell lines was compared with that of untreated control and fold change due to the treatment was expressed as 2^−ΔΔCt^ [61]. A 2.0-fold change in gene expression was used as the cut-off threshold.

### Statistical analysis

Statistical analyses were performed using Student's *t* test or one-way ANOVA (Dunnett's test) at a significance level of *p* < 0.05 (GraphPad Prism Software).

## References

[1] Fruman DA, Rommel C (2014). PI3K and cancer: lessons, challenges and opportunities. Nat Rev Drug Discov.

[2] Cantley LC (2002). The phosphoinositide 3-kinase pathway. Science.

[3] Bunney TD, Katan M (2010). Phosphoinositide signalling in cancer: beyond PI3K and PTEN. Nat Rev Cancer.

[4] Fayard E, Moncayo G, Hemmings BA, Hollander GA (2010). Phosphatidylinositol 3-kinase signaling in thymocytes: the need for stringent control. Sci Signal.

[5] So L, Fruman DA (2012). PI3K signalling in B- and T-lymphocytes: new developments and therapeutic advances. Biochem J.

[6] Barbee SD, Alberola-Ila J (2006). Phosphatidylinositol 3-kinase improves the efficiency of positive selection. Int Immunol.

[7] Hennessy BT, Smith DL, Ram PT, Lu Y, Mills GB (2005). Exploiting the PI3K/AKT pathway for cancer drug discovery. Nat Rev Drug Discov.

[8] Lunardi A, Webster KA, Papa A, Padmani B, Clohessy JG, Bronson RT, Pandolfi PP (2014). Role of aberrant PI3K pathway activation in gallbladder tumorigenesis. Oncotarget.

[9] Janku F, Kaseb AO, Tsimberidou AM, Wolff RA, Kurzrock R (2014). Identification of novel therapeutic targets in the PI3K/AKT/mTOR pathway in hepatocellular carcinoma using targeted next generation sequencing. Oncotarget.

[10] Bhojwani D, Pui CH (2013). Relapsed childhood acute lymphoblastic leukaemia. Lancet Oncol.

[11] Silva A, Yunes JA, Cardoso BA, Martins LR, Jotta PY, Abecasis M, Nowill AE, Leslie NR, Cardoso AA, Barata JT (2008). PTEN posttranslational inactivation and hyperactivation of the PI3K/Akt pathway sustain primary T cell leukemia viability. J Clin Invest.

[12] Palomero T, Sulis ML, Cortina M, Real PJ, Barnes K, Ciofani M, Caparros E, Buteau J, Brown K, Perkins SL, Bhagat G, Agarwal AM, Basso G, Castillo M, Nagase S, Cordon-Cardo C (2007). Mutational loss of PTEN induces resistance to NOTCH1 inhibition in T-cell leukemia. Nat Med.

[13] Silva A, Girio A, Cebola I, Santos CI, Antunes F, Barata JT (2011). Intracellular reactive oxygen species are essential for PI3K/Akt/mTOR-dependent IL-7-mediated viability of T-cell acute lymphoblastic leukemia cells. Leukemia.

[14] Subramaniam PS, Whye DW, Efimenko E, Chen J, Tosello V, De Keersmaecker K, Kashishian A, Thompson MA, Castillo M, Cordon-Cardo C, Dave UP, Ferrando A, Lannutti BJ, Diacovo TG (2012). Targeting nonclassical oncogenes for therapy in T-ALL. Cancer Cell.

[15] Stengel C, Jenner E, Meja K, Mayekar S, Khwaja A (2013). Proliferation of PTEN-deficient haematopoietic tumour cells is not affected by isoform-selective inhibition of p110 PI3-kinase and requires blockade of all class 1 PI3K activity. Br J Haematol.

[16] Trinquand A, Tanguy-Schmidt A, Ben Abdelali R, Lambert J, Beldjord K, Lengline E, De Gunzburg N, Payet-Bornet D, Lhermitte L, Mossafa H, Lheritier V, Bond J, Huguet F, Buzyn A, Leguay T, Cahn JY (2013). Toward a NOTCH1/FBXW7/RAS/PTEN-based oncogenetic risk classification of adult T-cell acute lymphoblastic leukemia: a Group for Research in Adult Acute Lymphoblastic Leukemia study. J Clin Oncol.

[17] Lonetti A, Antunes IL, Chiarini F, Orsini E, Buontempo F, Ricci F, Tazzari PL, Pagliaro P, Melchionda F, Pession A, Bertaina A, Locatelli F, McCubrey JA, Barata JT, Martelli AM (2014). Activity of the pan-class I phosphoinositide 3-kinase inhibitor NVP-BKM120 in T-cell acute lymphoblastic leukemia. Leukemia.

[18] Liu WL, Gao M, Tzen KY, Tsai CL, Hsu FM, Cheng AL, Cheng JC (2014). Targeting Phosphatidylinositide3-Kinase/Akt pathway by BKM120 for radiosensitization in hepatocellular carcinoma. Oncotarget.

[19] Rodon J, Brana I, Siu LL, De Jonge MJ, Homji N, Mills D, Di Tomaso E, Sarr C, Trandafir L, Massacesi C, Eskens F, Bendell JC (2014). Phase I dose-escalation and -expansion study of buparlisib (BKM120), an oral pan-Class I PI3K inhibitor, in patients with advanced solid tumors. Invest New Drugs.

[20] Ando Y, Inada-Inoue M, Mitsuma A, Yoshino T, Ohtsu A, Suenaga N, Sato M, Kakizume T, Robson M, Quadt C, Doi T (2014). Phase I dose-escalation study of buparlisib (BKM120), an oral pan-class I PI3K inhibitor, in Japanese patients with advanced solid tumors. Cancer Sci.

[21] Bendell JC, Rodon J, Burris HA, de Jonge M, Verweij J, Birle D, Demanse D, De Buck SS, Ru QC, Peters M, Goldbrunner M, Baselga J (2012). Phase, I, dose-escalation study of BKM120, an oral pan-Class I PI3K inhibitor, in patients with advanced solid tumors. J Clin Oncol.

[22] Yaguchi S, Fukui Y, Koshimizu I, Yoshimi H, Matsuno T, Gouda H, Hirono S, Yamazaki K, Yamori T (2006). Antitumor activity of ZSTK474, a new phosphatidylinositol 3-kinase inhibitor. J Natl Cancer Inst.

[23] Dan S, Yoshimi H, Okamura M, Mukai Y, Yamori T (2009). Inhibition of PI3K by ZSTK474 suppressed tumor growth not via apoptosis but G0/G1 arrest. Biochem Biophys Res Commun.

[24] Dan S, Okamura M, Mukai Y, Yoshimi H, Inoue Y, Hanyu A, Sakaue-Sawano A, Imamura T, Miyawaki A, Yamori T (2012). ZSTK474, a specific phosphatidylinositol 3-kinase inhibitor, induces G1 arrest of the cell cycle *in vivo*. Eur J Cancer.

[25] So L, Yea SS, Oak JS, Lu M, Manmadhan A, Ke QH, Janes MR, Kessler LV, Kucharski JM, Li LS, Martin MB, Ren P, Jessen KA, Liu Y, Rommel C, Fruman DA (2013). Selective inhibition of phosphoinositide 3-kinase p110α preserves lymphocyte function. J Biol Chem.

[26] Gilbert JA (2014). Idelalisib: targeting PI3Kδ, in B-cell malignancies. Lancet Oncol.

[27] Brown JR, Byrd JC, Coutre SE, Benson DM, Flinn IW, Wagner-Johnston ND, Spurgeon SE, Kahl BS, Bello C, Webb HK, Johnson DM, Peterman S, Li D, Jahn TM, Lannutti BJ, Ulrich RG (2014). Idelalisib, an inhibitor of phosphatidylinositol 3-kinase p110δ, for relapsed/refractory chronic lymphocytic leukemia. Blood.

[28] Flinn IW, Kahl BS, Leonard JP, Furman RR, Brown JR, Byrd JC, Wagner-Johnston ND, Coutre SE, Benson DM, Peterman S, Cho Y, Webb HK, Johnson DM, Yu AS, Ulrich RG, Godfrey WR (2014). Idelalisib, a selective inhibitor of phosphatidylinositol 3-kinase-δ, as therapy for previously treated indolent non-Hodgkin lymphoma. Blood.

[29] Winkler DG, Faia KL, DiNitto JP, Ali JA, White KF, Brophy EE, Pink MM, Proctor JL, Lussier J, Martin CM, Hoyt JG, Tillotson B, Murphy EL, Lim AR, Thomas BD, Macdougall JR (2013). PI3K-δ, and PI3K-γ inhibition by IPI-145 abrogates immune responses and suppresses activity in autoimmune and inflammatory disease models. Chem Biol.

[30] Desai AV, El-Bakkar H, Abdul-Hay M (2014). Novel Agents in the Treatment of Chronic Lymphocytic Leukemia: A Review About the Future. Clin Lymphoma Myeloma Leuk.

[31] (2014). IPI-145 shows promise in CLL patients. Cancer Discov.

[32] Weigel MT, Dowsett M (2010). Current and emerging biomarkers in breast cancer: prognosis and prediction. Endocr Relat Cancer.

[33] Buontempo F, Orsini E, Martins LR, Antunes I, Lonetti A, Chiarini F, Tabellini G, Evangelisti C, Melchionda F, Pession A, Bertaina A, Locatelli F, McCubrey JA, Cappellini A, Barata JT, Martelli AM (2014). Cytotoxic activity of the casein kinase 2 inhibitor CX-4945 against T-cell acute lymphoblastic leukemia: targeting the unfolded protein response signaling. Leukemia.

[34] Griner EM, Kazanietz MG (2007). Protein kinase C and other diacylglycerol effectors in cancer. Nat Rev Cancer.

[35] Evangelisti C, Chiarini F, Lonetti A, Buontempo F, Neri LM, McCubrey JA, Martelli AM (2014). Autophagy in acute leukemias: A double-edged sword with important therapeutic implications. Biochim Biophys Acta.

[36] Galluzzi L, Vitale I, Abrams JM, Alnemri ES, Baehrecke EH, Blagosklonny MV, Dawson TM, Dawson VL, El-Deiry WS, Fulda S, Gottlieb E, Green DR, Hengartner MO, Kepp O, Knight RA, Kumar S (2012). Molecular definitions of cell death subroutines: recommendations of the Nomenclature Committee on Cell Death. Cell Death Differ.

[37] Rambold AS, Lippincott-Schwartz J (2011). Mechanisms of mitochondria and autophagy crosstalk. Cell Cycle.

[38] Chen KL, Chang WS, Cheung CH, Lin CC, Huang CC, Yang YN, Kuo CP, Kuo CC, Chang YH, Liu KJ, Wu CM, Chang JY (2012). Targeting cathepsin S induces tumor cell autophagy via the EGFR-ERK signaling pathway. Cancer Lett.

[39] Zhang L, Wang H, Xu J, Zhu J, Ding K (2014). Inhibition of cathepsin S induces autophagy and apoptosis in human glioblastoma cell lines through ROS-mediated PI3K/AKT/mTOR/p70S6K and JNK signaling pathways. Toxicol Lett.

[40] Samuels Y, Wang Z, Bardelli A, Silliman N, Ptak J, Szabo S, Yan H, Gazdar A, Powell SM, Riggins GJ, Willson JK, Markowitz S, Kinzler KW, Vogelstein B, Velculescu VE (2004). High frequency of mutations of the PIK3CA gene in human cancers. Science.

[41] Tzenaki N, Papakonstanti EA (2013). p110δ, PI3 kinase pathway: emerging roles in cancer. Front Oncol.

[42] Wang X, Zhang X, Li BS, Zhai X, Yang Z, Ding LX, Wang H, Liang C, Zhu W, Ding J, Meng LH (2014). Simultaneous targeting of PI3Kδ, and a PI3Kδ, -dependent MEK1/2-Erk1/2 pathway for therapy in pediatric B-cell acute lymphoblastic leukemia. Oncotarget.

[43] Kang S, Denley A, Vanhaesebroeck B, Vogt PK (2006). Oncogenic transformation induced by the p110β, γ, and −δ, isoforms of class I phosphoinositide 3-kinase. Proc Natl Acad Sci U S A.

[44] Dbouk HA, Khalil BD, Wu H, Shymanets A, Nurnberg B, Backer JM (2013). Characterization of a tumor-associated activating mutation of the p110β PI 3-kinase. PLoS One.

[45] Li B, Sun A, Jiang W, Thrasher JB, Terranova P (2014). PI-3 kinase p110β: a therapeutic target in advanced prostate cancers. Am J Clin Exp Urol.

[46] Fruman DA, Cantley LC (2014). Idelalisib: a PI3Kδ, inhibitor for B-cell cancers. N Engl J Med.

[47] Vanhaesebroeck B, Ali K, Bilancio A, Geering B, Foukas LC (2005). Signalling by PI3K isoforms: insights from gene-targeted mice. Trends Biochem Sci.

[48] Foukas LC, Berenjeno IM, Gray A, Khwaja A, Vanhaesebroeck B (2010). Activity of any class IA PI3K isoform can sustain cell proliferation and survival. Proc Natl Acad Sci U S A.

[49] Costa C, Ebi H, Martini M, Beausoleil SA, Faber AC, Jakubik CT, Huang A, Wang Y, Nishtala M, Hall B, Rikova K, Zhao J, Hirsch E, Benes CH, Engelman JA (2014). Measurement of PIP_3_ Levels Reveals an Unexpected Role for p110β in Early Adaptive Responses to p110α-Specific Inhibitors in Luminal Breast Cancer. Cancer Cell.

[50] Schwartz S, Wongvipat J, Trigwell CB, Hancox U, Carver BS, Rodrik-Outmezguine V, Will M, Yellen P, de Stanchina E, Baselga J, Scher HI, Barry ST, Sawyers CL, Chandarlapaty S, Rosen N (2014). Feedback Suppression of PI3Kα Signaling in PTEN-Mutated Tumors Is Relieved by Selective Inhibition of PI3Kβ. Cancer Cell.

[51] Anderson NM, Harrold I, Mansour MR, Sanda T, McKeown M, Nagykary N, Bradner JE, Lan Zhang G, Look AT, Feng H (2014). BCL2-specific inhibitor ABT-199 synergizes strongly with cytarabine against the early immature LOUCY cell line but not more-differentiated T-ALL cell lines. Leukemia.

[52] Bohnacker T, Marone R, Collmann E, Calvez R, Hirsch E, Wymann MP (2009). PI3Kgamma adaptor subunits define coupling to degranulation and cell motility by distinct PtdIns(3,4,5)P3 pools in mast cells. Sci Signal.

[53] Gasser JA, Inuzuka H, Lau AW, Wei W, Beroukhim R, Toker A (2014). SGK3 Mediates INPP4B-Dependent PI3K Signaling in Breast Cancer. Mol Cell.

[54] Ouyang L, Shi Z, Zhao S, Wang FT, Zhou TT, Liu B, Bao JK (2012). Programmed cell death pathways in cancer: a review of apoptosis, autophagy and programmed necrosis. Cell Prolif.

[55] Mammucari C, Milan G, Romanello V, Masiero E, Rudolf R, Del Piccolo P, Burden SJ, Di Lisi R, Sandri C, Zhao J, Goldberg AL, Schiaffino S, Sandri M (2007). FoxO3 controls autophagy in skeletal muscle *in vivo*. Cell Metab.

[56] Zhao J, Brault JJ, Schild A, Cao P, Sandri M, Schiaffino S, Lecker SH, Goldberg AL (2007). FoxO3 coordinately activates protein degradation by the autophagic/lysosomal and proteasomal pathways in atrophying muscle cells. Cell Metab.

[57] Klauschen F, Andreeff M, Keilholz U, Dietel M, Stenzinger A (2014). The combinatorial complexity of cancer precision medicine. Oncoscience.

[58] Chou TC, Talalay P (1984). Quantitative analysis of dose-effect relationships: the combined effects of multiple drugs or enzyme inhibitors. Adv Enzyme Regul.

[59] Sparta AM, Bressanin D, Chiarini F, Lonetti A, Cappellini A, Evangelisti C, Melchionda F, Pession A, Bertaina A, Locatelli F, McCubrey JA, Martelli AM (2014). Therapeutic targeting of Polo-like kinase-1 and Aurora kinases in T-cell acute lymphoblastic leukemia. Cell Cycle.

[60] Evangelisti C, Teti G, Chiarini F, Falconi M, Melchionda F, Pession A, Bertaina A, Locatelli F, McCubrey JA, Beak DJ, Bittman R, Pyne S, Pyne NJ, Martelli AM (2014). Assessment of the effect of sphingosine kinase inhibitors on apoptosis, unfolded protein response and autophagy of T-cell acute lymphoblastic leukemia cells; indications for novel therapeutics. Oncotarget.

[61] Schmittgen TD, Livak KJ (2008). Analyzing real-time PCR data by the comparative C(T) method. Nat Protoc.

[62] Saeed AI, Bhagabati NK, Braisted JC, Liang W, Sharov V, Howe EA, Li J, Thiagarajan M, White JA, Quackenbush J (2006). TM4 microarray software suite. Methods Enzymol.

